# Enhancing Selenium Accumulation in *Rhodotorula mucilaginosa* Strain 6S Using a Proteomic Approach for Aquafeed Development

**DOI:** 10.3390/biom14060629

**Published:** 2024-05-27

**Authors:** Paola Díaz-Navarrete, Alberto Sáez-Arteaga, Luis Marileo, David Alors, David Correa-Galeote, Patricio Dantagnan

**Affiliations:** 1 Departamento de Ciencias Veterinarias y Salud Pública, Facultad de Recursos Naturales, Universidad Católica de Temuco, Temuco 4780000, Chile; 2 Núcleo de Investigación en Producción Alimentaria, Departamento de Ciencias Agropecuarias y Acuícolas, Facultad de Recursos Naturales, Universidad Católica de Temuco, Temuco 4780000, Chile; dantagna@uct.cl; 3 Centro de Investigación Innovación y Creación (CIIC-UCT), Universidad Católica de Temuco, Temuco 4780000, Chile; alberto.saez@uct.cl; 4 Departamento de Ciencias Agropecuarias y Acuícolas, Facultad de Recursos Naturales, Universidad Católica de Temuco, Temuco 4780000, Chile; 5 Escuela de Medicina Veterinaria, Facultad de Recursos Naturales y Medicina Veterinaria, Universidad Santo Tomás, Temuco 4780000, Chile; lmarileo@santotomas.cl; 6 Departamento de Ciencias Biológicas y Químicas, Facultad de Recursos Naturales, Universidad Católica de Temuco, Temuco 4780000, Chile; dalors@uct.cl; 7 Departamento de Microbiología, Facultad de Farmacia, Universidad de Granada, 18012 Granada, Spain; dcorrea@ugr.es

**Keywords:** *Rhodotorula mucilaginosa*, selenium-enriched yeasts, selenium

## Abstract

It is known that selenium (Se) is an essential trace element, important for the growth and other biological functions of fish. One of its most important functions is to contribute to the preservation of certain biological components, such as DNA, proteins, and lipids, providing protection against free radicals resulting from normal metabolism. The objective of this study was to evaluate and optimize selenium accumulation in the native yeast *Rhodotorula mucilaginosa* 6S. Sodium selenite was evaluated at different concentrations (5–10–15–20–30–40 mg/L). Similarly, the effects of different concentrations of nitrogen sources and pH on cell growth and selenium accumulation in the yeast were analyzed. Subsequently, the best cultivation conditions were scaled up to a 2 L reactor with constant aeration, and the proteome of the yeast cultured with and without sodium selenite was evaluated. The optimal conditions for biomass generation and selenium accumulation were found with ammonium chloride and pH 5.5. Incorporating sodium selenite (30 mg/L) during the exponential phase in the bioreactor after 72 h of cultivation resulted in 10 g/L of biomass, with 0.25 mg total Se/g biomass, composed of 25% proteins, 15% lipids, and 0.850 mg total carotenoids/g biomass. The analysis of the proteomes associated with yeast cultivation with and without selenium revealed a total of 1871 proteins. The results obtained showed that the dynamic changes in the proteome, in response to selenium in the experimental medium, are directly related to catalytic activity and oxidoreductase activity in the yeast. *R. mucilaginosa* 6S could be an alternative for the generation of selenium-rich biomass with a composition of other nutritional compounds also of interest in aquaculture, such as proteins, lipids, and pigments.

## 1. Introduction

Selenium is an essential trace element, important for growth and other biological functions in fish [1]. One of its primary functions involves safeguarding essential biological elements like DNA, proteins, and lipids by shielding them against free radicals, which arise as natural byproducts of cellular metabolism [2,3]. A low availability of this element can cause important physiological dysfunctions and can make cultivated fish more susceptible to some stressors, typical of confinement [4]. Selenium is essential for the functioning of the so-called selenoproteins, such as some seleno-dependent glutathione peroxidase isoforms, an enzyme which plays a crucial antioxidant role in reducing cellular damage generated by free radicals [5]. Selenium supplementation in the feed of farm animals, including fish, contributes to counteracting the problems caused by deficiencies of this element in captive conditions (decreased growth performance, increased rates of larval deformity, reduced rate of survival, an impaired immune system, dysfunction of endocrine glands, and antioxidant enzyme activity and imbalanced swimming and mortality), thus improving physiological responses when fish are subjected to conditions of constant environmental stress [6]. Sodium selenite (NaSe) is the most common and traditional source of selenium added in animal feed, including fish feed. In recent observations, it has been noted that diorganic selenium is more readily absorbed and exhibits greater potency in terms of bioavailability and health benefits in contrast to inorganic forms [6,7]. Thus, different sources of organic selenium are normally incorporated in animal feed, and their use has been widely accepted in animal nutrition [8]. The main way it is added to food is largely through the utilization of selenized yeasts [4] The accumulation of selenium in yeast biomass is carried out by a process of propagation in a medium containing this element, but only at low concentrations. If certain amounts of selenium are exceeded in the medium, it acts as an inhibitor of microbial metabolism, ultimately leading to the death of the yeast [9,10,11]. The sulfur in sulfur-containing amino acids in yeast cells is partially replaced by the selenium derived from inorganic sources [12]. After biochemical conversion, inorganic selenium in culture broth is converted to organic selenium in yeast cells. Selenium is enriched by yeast to form “Selenium yeast” (SY), which is a green feed additive [13]. To achieve this, an adequate mode of propagation is key, one that is able to guarantee high concentrations of biomass in a reactor while also regulating feeding of the nutrient of interest, so that it does not exceed the concentrations that inhibit or stop yeast growth [14]. Selenized yeasts, which predominantly contain seleniomethionine (Se-Met) and other compounds, are the most important sources of organic selenium used in animal production [2]. However, it has also been proposed that selenium in the form of nanoparticles may have an even greater benefit than solely administering organic or inorganic selenium compounds [4]. Indeed, beneficial properties have been demonstrated, such as lower toxicity and better yields compared to organic and inorganic selenium, using this approach [15,16]. Work related to the bioconversion of inorganic to organic selenium has mainly focused on the yeast *Saccharomyces cerevisiae*, as it is the main source of organic selenium in the nutrition of fish and other animals [7,13,17,18]. However, there have been several studies on other yeast genera, such as *Yarrowia* spp. [11], *Kluyveromyces* spp., and *Rhodotorula* spp. [19,20,21], in which the capacity for selenium accumulation in their cellular structure has also been demonstrated [22,23]. In recent years, SY supplementation has also been reported to enhance growth, feed conversion, and immunocompetence in channel catfish (*Ictalurus punctatus*), hybrid striped bass (*Moronechrysops Morone saxatilis*), rainbow trout (*Oncorhynchus mykiss*), and marron (*Cherax cainii*) [5].

Given the above, the objective of this study was to evaluate and optimize selenium accumulation in the native yeast *Rhodotorula mucilaginosa* 6S.

## 2. Materials and Methods

### 2.1. Collection of Soil Samples and Isolation of Yeast

The isolation of yeasts was conducted through serial dilution. Initially, 10 g of the respective soil samples (from the Araucanía Region at coordinates 38°42′08.1″ S 72°32′49.7″ W, North Campus University Catholic of Temuco) were weighed and placed in a flask. Subsequently, 90 mL of sterile distilled water was added, and the mixture was manually shaken until complete disaggregation of the soil was achieved. Then, 1 mL of this mixture was extracted using a sterile pipette and transferred to a tube containing 9 mL of sterile distilled water (10^−2^ dilution). Further dilutions were prepared up to 10^−6^ [24]. Next, 1 mL of the respective dilution was plated onto an empty Petri dish, to which 0.5 mL of an antibiotic mixture (penicillin and streptomycin in a 1:1 ratio) was added. The culture medium was adjusted to contain 60 mg/L of Na_2_SeO_3_ (Sigma Aldrich, St. Louis, MO, USA) (NaSe) (0.12 mL from a stock solution of 10 g/L of sodium selenite), and 18.38 mL of Sabouraud agar were added. After the agar had solidified, the plates were incubated at 23 ± 2 °C for seven days. Subsequently, the strain that did not exhibit growth inhibition was selected. For this purpose, 100 µL (0.5 McFaland) of the yeast under study were added to the center of the Petri dish, surface-seeded using a Digralsky loop. Subsequently, they were incubated at 22 ± 1 °C for 72 h. At the end of the incubation period, the colony-forming units per milliliter (UFC/mL) were counted.

### 2.2. DNA Extraction and Amplification of the ITS rDNA Region

The general characterization of the selected microorganism is described below: Molecular identification. The partial amplification of the 26S gene of nuclear ribosomal DNA (rDNA) was amplified using primers ITS1 (5′ TCC GTA GGT GAA CCT GCG G 3’) and NL4 (5’ GGT CCG TGT TTC AAG ACG G 3’) [25]. The PCR reaction was carried out in 25 μL containing GoTaq^®^ Green Master Mix (Taq DNA polymerase, dNTPs, MgCl_2_) (Promega, Madison, WI, USA) and 0.5 μL of the forward and reverse primers. Amplification of the ITS1-ITS4 fragment was performed under the following conditions: initial denaturation (2 min at 95 °C), 30 cycles of denaturation (94 °C for 1 min), annealing (40 s at 52 °C), and extension (1 min at 72 °C), followed by a final extension (10 min at 72 °C). Four μL of the PCR product were analyzed on 1.5% TAE 1X agarose gels and visualized using a FOTO/UV21 Transilluminator Haverhill, MA, USA. A 100 bp DNA molecular weight marker (Thermo Fisher Scientific Inc., Waltham, MA, USA) was used [25]. The DNA concentration was measured on a Nanodrop, Infinite M200. The amplified DNA was purified and sent for sequencing to Austral-Omics, Valdivia-Chile.

### 2.3. Molecular Identification and Phylogenetic Analysis

The nuclear ITS sequence of the isolated *Rhodotorula* sp. strain (herein after named *Rhodotorula mucilaginosa* 6S) was compared with the ITS sequences in the NCBI repository using the Basic local alignment search tool “BLAST” [26]. The sequence was 100% identical to those identified as *R. mucilaginosa* 6S. To establish the phylogenetic position, the sample was compared with 12 type species of Rhodotorula and 2 other species which were used as outgroups [27,28]. The sequences were aligned using MUSCLE in MEGA; the resulting DNA matrix was then manually revised, and nucleotide insertions present in only one or two samples were removed. A DNA matrix of 591 bp was constructed after trimming the flanking regions of reference sequences to the size of the sequence identified. The nucleotide substitution model (T92+G) was estimated in MEGA and used for Bayesian analysis. Maximum likelihood analysis was performed using the RaxML [29] Cipres portal (www.Phylo.org), accessed on 17 January 2024. The Bayesian phylogenetic analysis was carried out using MrBayes [30] in the Cipres portal with 100,000 generations, sampling a tree each 100 generations and discarding the first 250 trees.

### 2.4. Preparation of the Inoculum and Strain Maintenance

The yeast was kept on solid medium at 4 °C in Sabouraud Agar and replicated every 4 weeks to create fresh medium. The yeast culture was stored in a cryopreserver at −80 °C after frozen in liquid nitrogen. The inoculum was prepared in liquid medium containing 2% glucose, 2% peptone, and 1% yeast extract, in a final volume of 100 mL [31]. The medium was inoculated with a fresh culture of yeast cultured for 24 h with cells in exponential phase at a concentration of 1 × 10^6^ cells/mL. The cultures were placed in a reciprocal shaker at a temperature of 25 °C and 150 rpm until the exponential phase was reached.

### 2.5. Effect of Sodium Selenite Concentration on Selenium Accumulation and Cell Growth

The experiments tested eight concentrations of NaSe (5–40 mg Se^4+^/L) (Sigma Aldrich) in the culture medium. Growth kinetics were determined for each concentration of selenium by taking samples after 6, 12, or 24 h (depending on the growth rate) and centrifuging at 4000 rpm for 20 min to remove the inorganic selenium (Eppendorf^®^ Centrifuge 5702 Hamburg, Germany); they were then washed three times in distilled water, and centrifugation was repeated. The response variables were analyzed in this biomass, using *R. mucilaginosa* 6S culture without NaSe in the culture medium as a negative control. The culture conditions were 25 °C, 150 rpm (Shaker, Benchtop model TOU-120-2 MRC Laboratory Instruments Ltd. Hahistadrut, Holon, Israel) in 500 mL flasks with a useful volume of 300 mL, from an initial inoculum (overnight culture) at 5% *v*/*v* (1 × 10^6^ cells/mL), for 5 d. All experiments were carried out in triplicate under axenic conditions.

### 2.6. Effect of pH and Nitrogen Source on Selenium Accumulation and Cell Growth

To evaluate environmental factors such as pH and nitrogen source, the following study ranges were established: pH: 4.5–5.5–6.5; nitrogen source: peptone, (NH_4_)_2_SO_4_, and NH_4_Cl. The selenium concentration used in the culture medium was 10 mg/L Na_2_SeO_3_. The experimental design employed for the analysis of these variables was a factorial experiment in triplicate. A basic culture medium (BCM) was used, composed of 3% glucose, 1% nitrogen (varied according to analysis), and 0.2% yeast extract. The pH was adjusted with 0.1 M HCl and 0.1 M NaOH, as appropriate. Previously, the yeast strains were inoculated in 100 mL of BCM for 36 h at 150 rpm and 25 °C (pre-culture). This pre-culture, adjusted to 10^6^ cells/mL, was suspended at 5% (*v*/*v*) in 300 mL of BMC and incubated at 200 rpm at 25 °C for 5 d. After that time, it was centrifuged at 5000 rpm for 20 min and washed three times with distilled water. Biomass was determined by weighing on an analytical balance and expressed as cell dry weight (g/L) according to [32].

### 2.7. Optimization in a 2 L Bioreactor and Nutritional Characterization of Biomass

In the 2 L bioreactor (INNOVA INOBIO-5G JG, TCL group), the optimal conditions for growth and selenium accumulation, were evaluated. The culture medium contained 30 g/L glucose, 10 g/L ammonium chloride, and 2 g/L yeast extract. The inoculum was previously incubated for 24 h to a concentration of 10^6^ cells/mL. Subsequently, two strategies for incorporating NaSe into the yeast were analyzed to enhance selenium accumulation and improve the nutritional characteristics of the biomass [18,33]. Strategy 1: Inoculation with NaSe (30 mg/L) at the beginning of the exponential phase: Yeast was cultured for 96 h (stationary phase) without adding NaSe to the culture medium. Subsequently, the biomass was harvested and washed three times with sterile deionized water by centrifugation at 4000 rpm for 20 min. Response variables were then analyzed. Strategy 2: Inoculation with NaSe (30 mg/L) at the end of the exponential phase: Yeast was cultured until the end of the exponential growth phase without NaSe in the culture medium (48 h). After this time, NaSe was added to a concentration of 30 mg/L in the culture medium, and the culture was continued until the stationary phase (72 h). Subsequently, the biomass was harvested and washed three times with sterile deionized water by centrifugation at 4000 rpm for 20 min, after which the response variables were analyzed. The cultivation conditions in the bioreactor were set at 150 rpm, with a 5% (*v*/*v*) inoculum, controlling foam formation (antifoam agent), air flow (1.5 L/min) and pH (5.5, using NaOH). The experiments were conducted in triplicate. The negative control was the culture of *R. mucilaginosa* 6S without selenium in the culture medium.

### 2.8. Protein Extraction and Analysis of Extracellular Fractions

Protein extraction from *R. mucilaginosa* 6S cells was carried out by adding 200 μL lysis buffer containing 8 M Urea, 100 mM NaCl, 50 mM ammonium bicarbonate pH 8.5, and 1X protease inhibitor (Complete Protease Inhibitor Cocktail, Roche, Lewes, UK). Subsequently, the samples were lysed using a sonicator for 10 cycles of 10 s each. Each sample was centrifuged at 15,000× *g* for 15 min and the pellet was separated from the proteins contained in the supernatant. Proteins were quantified using the Dual-Range™ BCA Protein Assay kit (Energenesis Biomedical Co., LTD, Taipei city, Taiwan), following the manufacturer’s instructions. Subsequently, samples were denatured with 2 M Urea and 50 mM ammonium bicarbonate (BICAM) pH 8, and the cysteines were reduced using 20 mM DTT at 60 °C for 30 min and then alkylated with 40 mM IAA for 15 min in the dark at room temperature. Finally, trypsin was added in a ratio of 1/50 (enzyme/substrate) and incubated overnight at 37 °C with shaking. Peptide extraction was performed with 80% acetonitrile and 0.1% TFA. The eluted peptides were dried by centrifugation using a SpeedVac (Thermo Fisher Scientific) for 30 min.

Peptide separation was performed with an Easy-nLC II liquid chromatograph coupled to a Q Exactive plus mass spectrometer (Thermo Fisher Scientific). Peptides were resolved using a C18 PepMap™ Easy-Spray reversed-phase column (75 µm × 15 cm) with a 3 µm particle size in a gradient of 6–35% acetonitrile for 30 min at a constant flow of 200 nL/min. Analysis parameters were set using Xcalibur™ acquisition software (version 4.2). The spectra were obtained using an Orbitrap analyzer in Full MS mode in a range of 300–1800 *m*/*z* and a resolution of 70,000. The 20 most intense ions were fragmented and the dd-MS2 spectra were acquired with a resolution of 17,500. The target value was set to 1 × 10^5^ with an isolation window of 1.8 *m*/*z*. The maximum injection time was 100 ms with a normalized collision energy of 28 eV.

The fragmentation spectra obtained were analyzed using the PEAKS^®^ Studio program (version 10.6) by searching the UniProt database restricted to *Rhodotorula* spp., with a parental/fragmented mass error tolerance of 10 ppm/0.02 Da. Possible false positives were filtered using a False Discovery Rate (FDR) of 1%. Variable modifications of the proteome were evaluated for oxidation of methionine, the conversion of glutamic acid to Pyro-glu, and glutamine to Pyro-glu (N-terminal), and deamination of asparagine and glutamine. In addition, a Label-Free Quantification (LFQ) analysis was performed with a mass error tolerance of 20 ppm and an FDR of 1% to determine significant differences between proteins identified in the control and treated conditions.

#### Proteome Data Processing

Protein raw data were filtered and contaminating fragments were removed. Using the PEAKS^®^ Studio analysis software (version 10.6), the area value was determined, which allowed the abundance of each protein to be calculated. Data with at least 2 areas for each group (control and treatment) were considered. The values were quantified and differentially expression determined using Perseus software (v2.0.10.0).

### 2.9. Analytical Methods

Quantification of cell biomass (yeast): Post-culture yield of yeast cell biomass was determined after centrifuging in a previously weighed thimble (3000× *g*, 10 min, 4 °C). Centrifuged cell biomass was dried at a temperature of 80 °C (WTC Binder oven, Tuttlingen, Germany) for 24 h to a constant weight. Biomass yield was expressed in grams of yeast dry weight (g d.w.) per liter of culture medium (g d.w.L^−1^ of medium). Determination of total selenium (ICP-OS): 0.5 g samples of biomass (yeast) were homogenized and mineralized with acid digestion using a mix of 65% nitric acid, 70% perchloric acid, 95–97% sulfuric acid, and 37% hydrochloric acid for 12 h at room temperature in a 100-mL flask. Subsequently, digestion at 220 °C was conducted for 3 h on a hot plate (Quimis Aparelhos Scientific Ltda. 6313 f22, São Paulo, Brazil). The resulting solution was diluted in a calibrated flask to 15 mL with ultrapure water. An ICP-OES (Optima 5300, Perkin Elmer, Norwalk, CT, USA) was used for selenium determination by the hydride generation technique. Fatty acid composition: The total lipid content was determined using a chloroform/methanol (2:1) mix [34]. Fatty acids were methylated following the method proposed by [35] and separated using a Hewlett Packard 589 series II Plus gas chromatograph (Wilmington, NC, USA) with a capillary column of 30 m × 0.25 mm × 0.20 µm (SPTM 2380, SUPELCO, Bellefonte, PA, USA). Helium gas was used as a transporting agent. The fatty acids were identified by comparing them with a SUPELCO 37 fatty acid reference standard (Sigma Aldrich, St. Louis, MO, USA). Fatty acids were expressed as a percentage of all identified fatty acids (wet basis) [35]. Total protein: The determination of total nitrogen was performed using the Kjeldahl method in a heated digester (DK20, Velp Scientifica, Usmate, Italy) with an automatic distillation unit (UDK 149, Velp Scientifica, Usmate, Italy), and protein was calculated using the factor N × 6.25. Extraction pigments were identified according to Garcia-Cortes et al. [36].

### 2.10. Statistical Analysis

To determine significant differences between the different conditions evaluated in this study, an ANOVA analysis was performed (*p* < 0.05), followed by Tukey’s post hoc test. Principal component analysis (PCA) was used to visualize the relationship between the treatments and the parameters evaluated. PRIMER v6 software (PRIMER–E Ltd., Auckland, New Zealand) was employed to analyze the PCA data. The ANOVA test, Tukey test, and Pearson’s correlation were performed using SPSS software version 26 (IBM, Armonk, NY, USA).

Student’s *t*-test was applied to determine if there were statistically significant differences in protein expression between the two conditions studied, considering a fold change rate ≥ 2 and a *p*-value ≤ 0.05. Subsequently, proteins with areas equal to 0 were eliminated to perform the ANOVA statistical test, which was represented by generating a heatmap. On the other hand, protein signaling pathways that presented significant differences were determined using the STRING platform.

## 3. Results

### 3.1. Identification of the Selected Yeast

**Figure 1:**
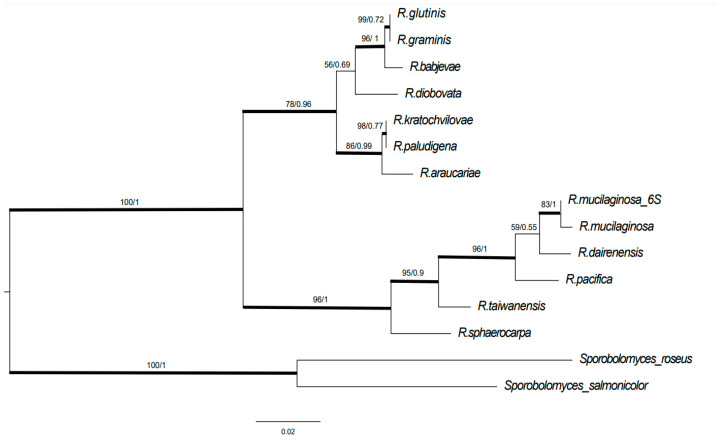
Maximum likelihood phylogenetic tree of *Rhodotorula* type species for the ITS region (ITS1-5.8S-ITS2). The *Sporobolomyces roseus* and *Sporobolomyces salmonicolor* sequences were used as outgroup. Bolded branches possess bootstrap values equal or higher than 70 and are considered to be statistically supported. Values on branches represents bootstrap support (left) and posterior probability (right).

After incubation, six yeast strains grew on agar with a high concentration of sodium selenite. Strain 6S presented abundant colony growth, unlike the other five strains that presented growth inhibition; strain 6S was thus selected for further study. The comparison with the GenBank database from the NCBI indicated that the sample belongs to *R. mucilaginosa* 6S, an identification that was supported by the phylogenetic position in both the maximum likelihood (ML) tree (Figure 1) and the Bayesian inference tree (Supporting Figure S1). Only two nucleotides were different in the whole ITS sequence between the reference sequence of *R. mucilaginosa* and the *Rhodotorula* strain isolated here. In the ML tree, strain 6S forms a clade with the sequence type of *R. mucilaginosa* (bootstrap support of 83) and with *R. dairenensis* and *R. taiwanensis*. Similarly, in the Bayesian tree generated by MrBayes, *R. mucilaginosa* 6S and the *R. mucilaginosa* type were grouped together with a posterior probability of 1, and with *R. dairenensis* and *R. pacifica* with a value of 1. In both phylogenetic trees, the *Rhodotorula* species were grouped in two robustly different clades, suggesting structure within the *Rhodotorula* genus. *R. mucilaginosa* was grouped not only with *R. dairanensis* and *R. pacifica* but also with *R. taiwanensis* and *R. sphaerocarpa*.

### 3.2. Effect of Sodium Selenite Concentration

**Figure 2:**
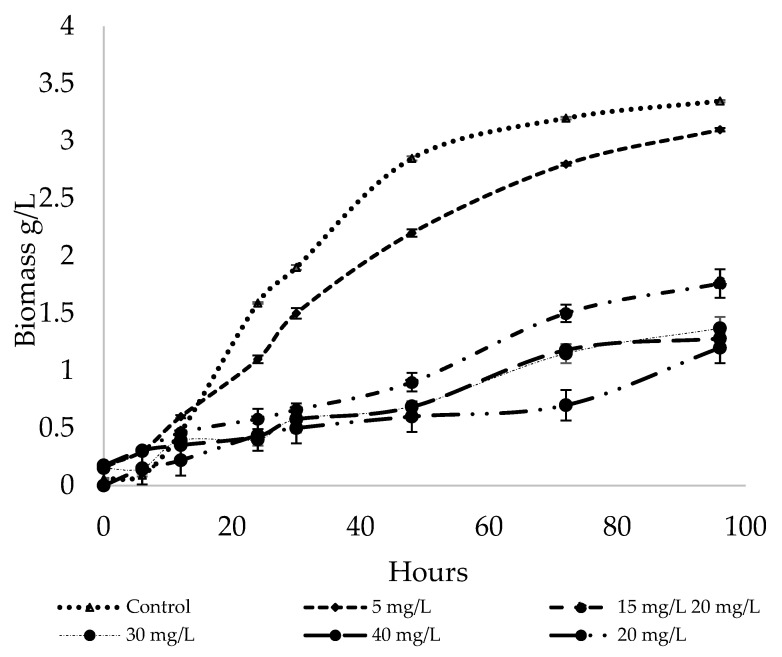
The growth kinetics of the yeast *R. mucilaginosa* 6S cultivated with different concentrations of NaSe.

The first assay conducted in this study involved analyzing the effect of the concentration of NaSe incorporated into the culture medium at the beginning of *R. mucilaginosa* 6S cultivation. Cell growth was monitored for five days (Figure 2). After 96 h of cultivation, it was determined that concentrations higher than 10 mg NaSe/L in the culture medium resulted in approximately a 48% reduction in total biomass compared to the control. For the control culture, a specific growth rate (µ) of 0.144 h^−1^ was obtained. Starting from 15 mg/L NaSe in the culture medium, µ (h^−1^) was considerably reduced to 0.0173, and at 40 mg/L NaSe, it fell even further to 0.0125. *R. mucilaginosa* has been reported to have µ (h^−1^) rates in the range from 0.2 to 0.29 h^−1^ using glucose as a carbon source [37,38]. Differences in these values, compared to the control, previously described could be associated with intrinsic characteristics of this native strain. Selenium is a mineral that induces metabolic stress (in the yeast), and therefore, the reduction in µ is directly related to the increase in NaSe concentration in the culture medium, as evidenced in this study.

**Figure 3:**
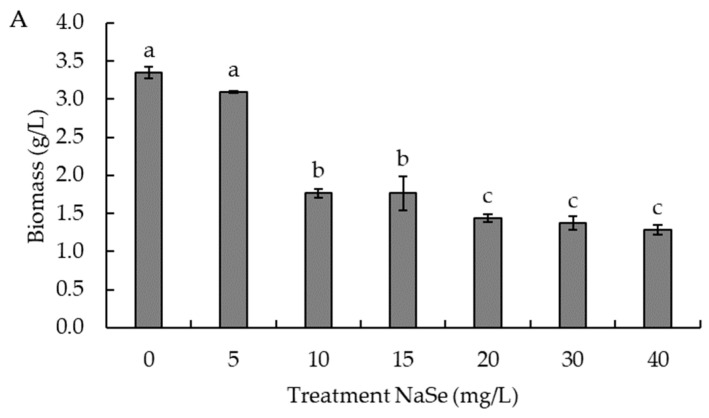

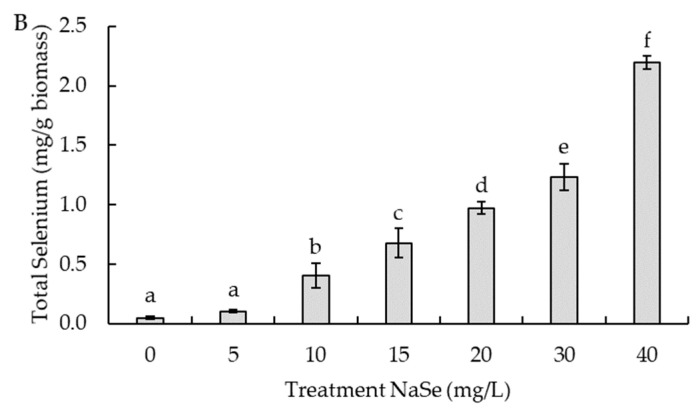
Effect of sodium selenite (NaSe) on (**A**) = biomass yield (g/L) and (**B**) = bioaccumulation of total selenium in biomass (mg/g biomass) in *R. mucilaginosa* 6S culture. Different letters represent significant differences between NaSe treatments with *p*-value < 0.05. Error bars represent SD.

As the concentration of NaSe increased in the culture medium, an inverse relationship with biomass generation is observed. As shown in Figure 3A, the NaSe treatments that generated significantly (*p* < 0.05) greater biomass production were those with 0 and 5 mg/L NaSe in the culture medium (3.3 and 3.1 g/L, respectively), while the treatments that generated significantly (*p* < 0.05) lower biomass production were treatments with 20, 30, and 40 mg/L NaSe in the culture medium (1.4, 1.4, and 1.3 g/L biomass, respectively).

Contrary to the biomass, the content of total selenium bioaccumulated in the yeast biomass (Figure 3B) increased as NaSe rose in the treatments performed. The conditions that generated the highest bioaccumulation of total selenium was the treatment with 40 mg/L NaSe, with a bioaccumulated selenium content of 2.2 ± 0.1 mg/g biomass.

### 3.3. Effect of pH and Nitrogen Source on Growth and Total Selenium Accumulation

**Figure 4:**
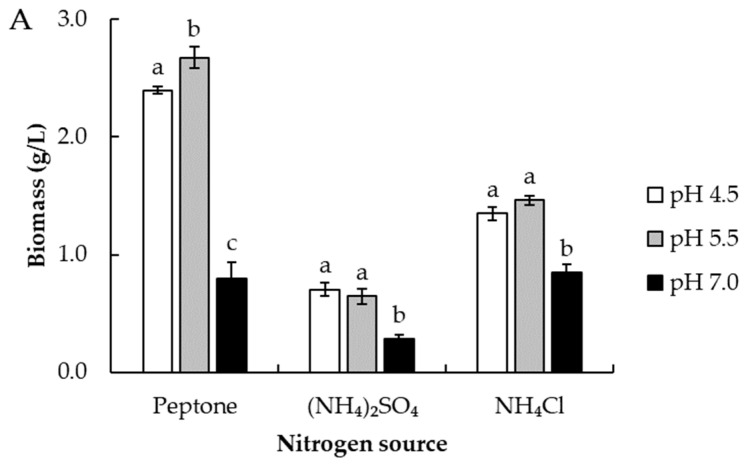

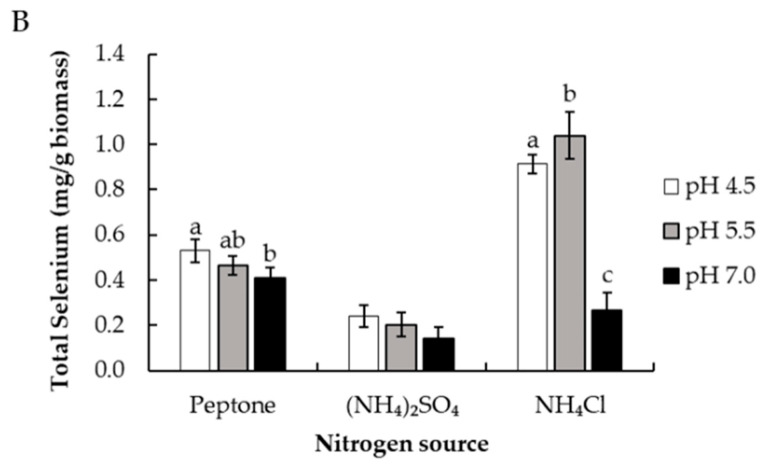
Effect of pH and nitrogen source (peptone; ammonium sulfate and ammonium chloride) on cell growth (**A**) and total selenium accumulation (**B**) in *R. mucilaginosa* 6S yeast. Error bars represent SD Different letters represent significant differences between NaSe treatments with *p*-value < 0.05.

Regarding the effect of the nitrogen source used in the culture medium together with the adjusted initial pH on biomass (Figure 4A), it was observed that the use of peptone in the culture medium generated the highest biomass yields (g/L) compared to ammonium sulfate and ammonium chloride. Moreover, the combination of peptone and low adjusted pH was an important interaction of variables in the biomass yield of *R. mucilaginosa* 6S. The highest biomass yields corresponded to the treatments using peptone as a nitrogen source and with the pH adjusted to 4.5 and 5.5 (2.4 and 2.6 g/L biomass, respectively), with a total bioaccumulation of selenium (Figure 4B) of 0.53 and 0.54 mg/g biomass. The highest bioaccumulation of total selenium was achieved with the use of ammonium chloride at an adjusted pH of 4.5 and 5.5, where total selenium (Figure 4B) reached 0.9 and 1.0 mg/g of biomass, respectively, with a biomass production (Figure 4A) of 1.4 and 1.5 g/L, respectively, for these treatments.

### 3.4. Cultivation in a 2 L Reactor and Biomass Characterization

**Figure 5:**
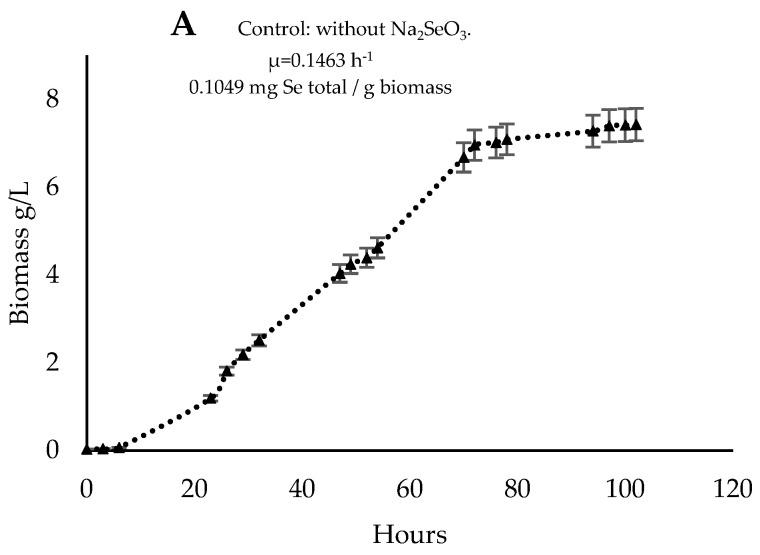

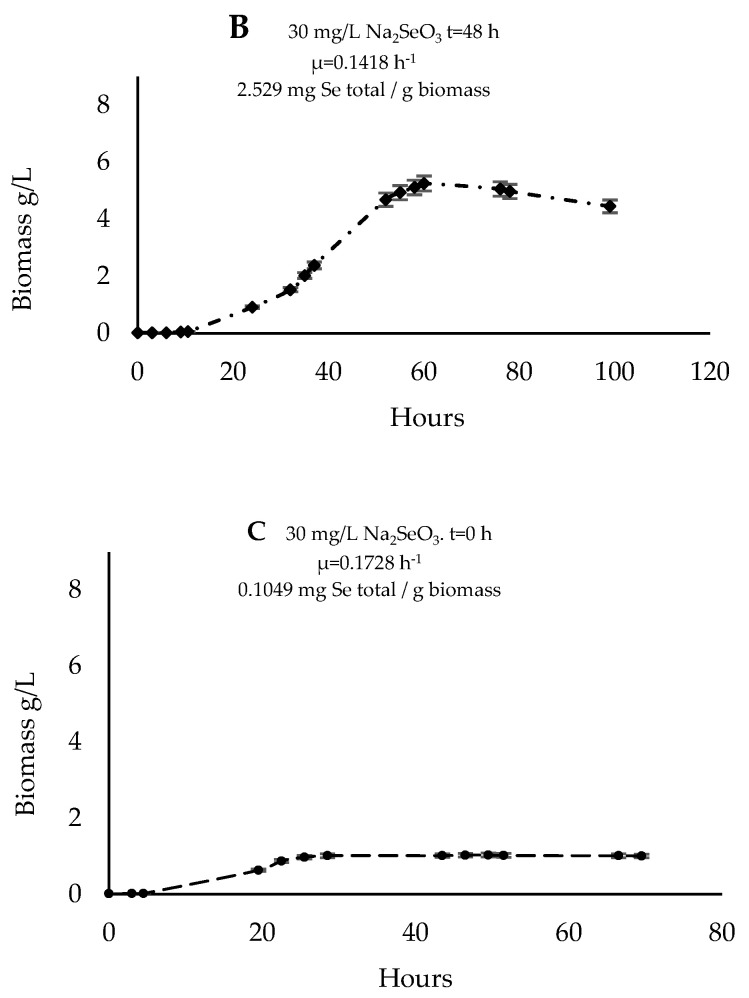
Growth kinetics of the yeast *R. mucilaginosa* 6S with two cultivation strategies. (**A**) Control: yeast cultivation without sodium selenite. (**B**) Yeast cultured with the incorporation of NaSe at 48 h. (**C**) Yeast cultured with the addition of sodium selenite at the beginning of the culture, t = 0. Error bars represent SD.

The growth kinetics of *R. mucilaginosa* 6S yeast cultivated in 2 L reactors with a concentration of 30 mg/L NaSe under constant aeration for five days was evaluated. In Figure 5A, it can be observed that the culture without Na_2_SeO_3_ resulted in a growth rate (µ) of 0.1463 h^−1^, reaching the exponential phase after 72 h of cultivation with a final biomass after 96 h of 7.42 g/L, a Yx/s (substrate yield) of 0.655, and 0.103 mg Se/g biomass. Figure 5B shows that the exponential phase was reached after 60 h of cultivation, with the growth rate (µ) decreasing after adding NaSe at 48 h of cultivation. At the end of the cultivation, 4.45 g/L was obtained with a Yx/s of 0.440 and a concentration of 2.529 mg Se/g biomass. When NaSe was incorporated at t = 0 (start of cultivation), as shown in Figure 5C, the exponential phase culminates after around 28 h with a µ of 0.1728 h^−1^, yielding 1 g/L of biomass after 96 h of cultivation with a concentration of 1 mg Se/g of biomass.

When evaluating the effect of the time (0 and 48 h after starting the culture) of NaSe addition (30 mg/L) on different productive parameters, it was observed (Table 1) that the addition of NaSe 48 h after starting the culture significantly increased (*p* < 0.05) the total lipid content (4.5%), the monounsaturated fatty acid (MUFA) content (69.5%), and the bioaccumulation of total selenium (2.5 mg/g biomass) with respect to the control (3.2% of total lipid, 24.0 of MUFA, and 0.1 mg/g biomass of total selenium).

**Figure 6:**
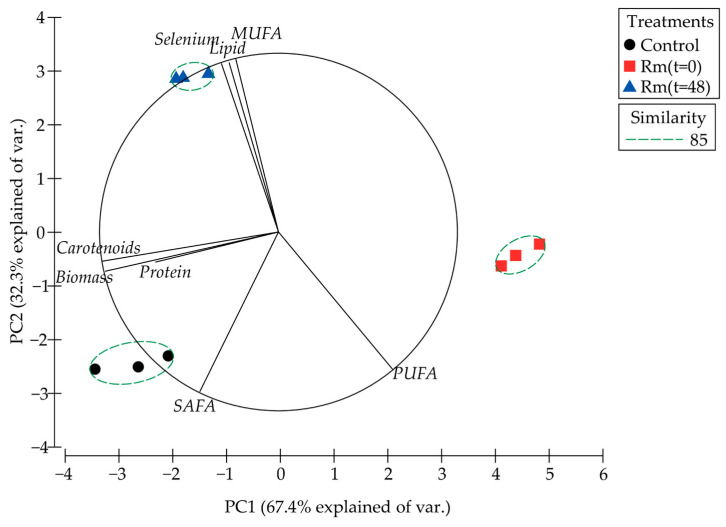
Principal component analysis (PCA) based on NaSe addition time treatments and the parameters evaluated. The similarity test grouped treatments by Bray–Curtis similarity.

Principal component analysis (PCA) of NaSe addition time treatments and the parameters evaluated (Figure 6) explained 99.7% of the total variance, where the first component (67.4%) was associated with biomass (g/L), protein (%), and carotenoids (µg/g biomass), and the second component (32.3%) was composed of total lipid (%), Saturated Fatty Acids (SAFA; %), MUFA (%), Poly-unsaturated Fatty Acids (PUFA; %), and bioaccumulation of total selenium (mg/g biomass). This PCA shows that the addition of NaSe at 48 h (quadrant II) after starting the culture presented a strong direct correlation with the bioaccumulation of total selenium, as well as the content of total lipids and MUFA, whereas this same treatment had a strong inverse correlation with the content of PUFA contained in the dry biomass of *R. mucilaginosa* 6S under these cultivation conditions.

Table 2 shows that the treatments presented a significant direct Pearson correlation (*p* < 0.05) with the total lipid content (r = 0.9), the percentage of MUFA (r = 0.9), and bioaccumulation of total selenium (r = 0.8), and a significant inverse correlation with the percentage of saturated fatty acids (r = –0.9). Furthermore, selenium bioaccumulation is a variable that was strongly and significantly correlated with the content of total lipids (r = 0.9) and the content of MUFA (r = 0.9), and was significantly inversely correlated with the content of PUFA (r = –0.9) under these growing conditions.

### 3.5. Proteome R. mucilaginosa 6S

**Figure 7:**
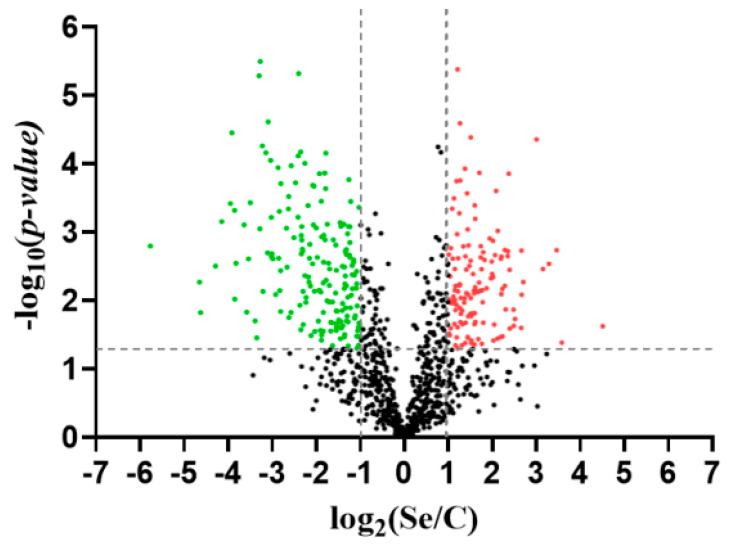
Changes in protein abundance due to the effect of selenium in *Rhodotorula* spp. The red dots indicate the proteins that were more abundant in the treatment with Se (133), and the green indicate those that were less abundant due to the treatment (178), or that were more abundant in the control. Vertical dashed lines indicate a fold change ≥ 2. The horizontal line indicates the significance that corresponds to a *p*-value ≤ 0.05. C: control, Se: samples treated with selenium.

Following the treatments and analyses detailed in Materials and Methods, 1886 proteins in total were initially detected. After identifying and eliminating contaminating proteins, the number of total proteins was reduced to 1871. Once the contaminant-free matrix was obtained, the data were filtered considering at least two areas for each group, reducing the total number of quantifiable proteins to 1002. Statistically significant differences (fold change ≥ 2, *p*-value ≤ 0.05) were detected in 311 proteins, of which 133 were upregulated in the treatment with Se, while 178 were less abundant due to the effect of the treatment, or they were more abundant in the control (Figure 7).

**Figure 8:**
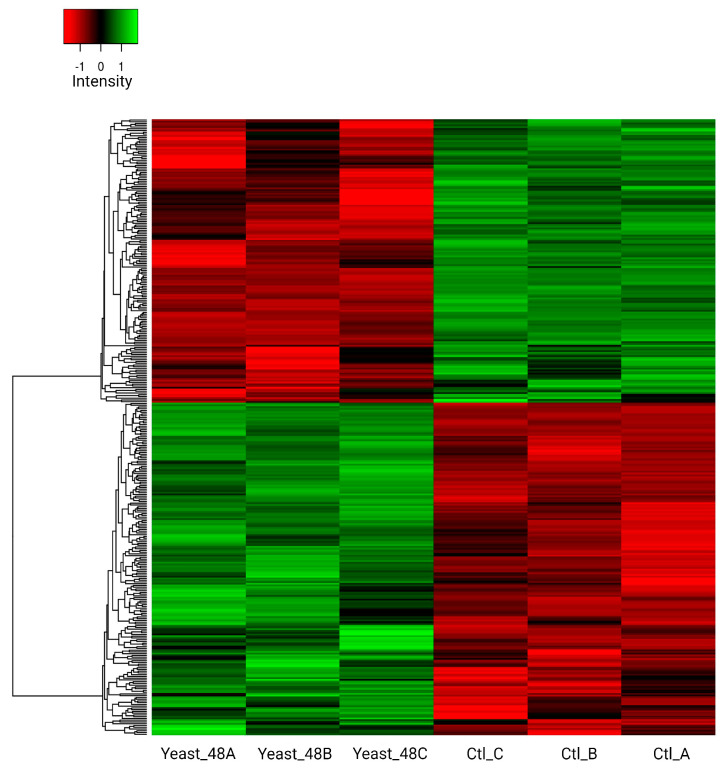
Heat map showing the abundance of 339 differentially present *R. mucilaginosa* 6S proteins identified from proteomic analysis in three replicates of control (Ct) and samples treated with NaSe for 48 h (48Se). The color scale illustrates the relative abundance of each protein in the 3 samples; red and green indicate higher and lower abundance compared to the median expression value (black), respectively. The intensity of the color indicates the degree of positive or negative regulation of the proteins.

The proteins differentially detected were analyzed in terms of STRING interaction networks to find potentially modulated global metabolic pathways. Of the 133 proteins that were more abundant in the selenium treatment, 71 were identified in the database available for *Rhodotorula* sp. CCFEE5036. Seven molecular functions were identified, of which the most predominant were proteins associated with catalytic activities and ion binding (44 and 31 proteins, respectively). On the other hand, the interaction of 126 proteins was determined in the control sample, in which proteins involved in only two molecular functions were identified, corresponding to catalytic activity and oxidoreductase activity (Figure S1 of Supplementary Materials). Hierarchical clustering analysis of proteins that exhibited differential abundance confirms a marked separation between the two experimental groups (Figure 8).

## 4. Discussion

*Rhodotorula mucilaginosa* is a well-known yeast of biotechnological significance that has attracted substantial attention as a prospective platform strain because of its varied appetites for substrates, strong stress tolerance, and other favorable characteristics. *R. mucilaginosa* is considered a great option for the biorefinery of carotenoids, lipids, enzymes, and other useful bioproducts, using inexpensive agricultural waste materials as substrates [39,40]. Ponce de León et al. [18] analyzed the biotransformation capacity of NaSe IV to selenomethionine (SeMet) in the yeast *Saccharomyces cerevisiae*, finding that the best method of obtaining a yeast rich in organic selenium was by adding low doses of NaSe (from 10 to 50 mg/L) in the exponential growth phase. The absorption of selenium by *Saccharomyces uvarum* was described by Marinescu and Stoicescu [41], who found that if this species was cultured for 24 h in a malt must with NaSe at a concentration between 30 and 180 μg/mL, a selenized biomass was obtained with large quantities of selenium, ranging between 625 and 2215 μg/g of dry yeast. The same was reported by Schrauzer [42], who established that, depending on the culture conditions and the strain, yeasts can accumulate large quantities of this element, up to 3000 μg/g of dry yeast. Similarly, Sun et al. [43], using a culture medium supplemented with 30 μg/mL NaSe added during the exponential growth phase, managed to accumulate selenium in the range of 1200–1400 μg/g of *S. cerevisiae*, as measured by the ICP-AES method. On the other hand, Kieliszek [11] assessed the effect of different concentrations of NaSe IV (10, 20, 40, and 60 mg Se^4+^/L) on the yeasts *S. cerevisiae* ATCC MYA-2200 and *Candida utilis* ATCC 9950, finding that the maximum production of biomass rich in organic selenium was obtained with *C. utilis* ATCC 9950 (15 g/L) after 24 h of culturing in 10 mg Se^4+^/L. When the culture medium was analyzed, which contained 60 mg Se^4+^/L, an amount of 5.64 mg Se^4+^/g dry yeast was detected in *S. cerevisiae* ATCC MYA-2200 after 72 h of culturing; for *C. utilis* ATCC 9950, under similar culture conditions, a quantity of 5.74 mg Se^4+^/g dry yeast was determined. Therefore, according to these results, yeasts of the genus *Candida* are more efficient at binding this element.

Subsequently, Kieliszek et al. 34] optimized culture conditions in a bioreactor with *C. utilis* ATCC 9950, obtaining 1841 μg Se/g dry yeast after 48 h of culturing with a mineral medium containing a concentration of 30 mg Se^4+^/L. In the present study, the concentration of NaSe had a direct impact on the biomass of *R. mucilaginosa* 6S. It was determined that values above 10 mg/L NaSe in the culture medium tended to inhibit cellular growth. At a concentration of 40 mg/L, although little biomass was obtained (0.48 g/L), over 2000 μg Se/g of yeast was accumulated.

Selenium induces metabolic stress in yeast by affecting cellular processes, leading to alterations in cell growth. The incorporation of selenium into the culture medium yields varied effects, dependent on the method employed [44]. Selenite and selenide cause death in yeast cells in a dose-dependent manner [44,45]. Selenate is also toxic for yeast cells, although considerably less than selenite when equivalent concentrations are compared for effects on cell viability, reactive oxygen species (ROS) production, or DNA damage. At minimal doses, cellular adaptation to enhance resistance occurs. Conversely, higher doses prompt the activation of various antioxidant functions mediated by the gene expression of Yap1p and Msn2,4p transcription factors, and cell-division cycle delay [46]. Selenium is recognized as a key element that acts as a cofactor and coenzyme in the catalytic-active sites of numerous Selenoproteins and enzymes, protecting cells from oxidative damage and stress. Selenium is important in selenoenzymes such as GPXs (glutathione peroxidase), TXNRDS (thioredoxin reductases), and DIO (deiodinases), contributing to a variety of biochemical events and physiological antioxidant defense systems. Selenium-enriched yeast cells exhibit changes in fatty acid profiles, with an increase in unsaturated fatty acids and alterations in amino acid content, impacting the integrity of the cytoplasmic membrane [47,48]. Additionally, selenium accumulation in yeast structures, production of selenium nanoparticles, and the formation of Se compounds combined with glutathione further contribute to the metabolic stress induced by Selenium [49]. Overall, selenium’s high dose presence in yeast cultures leads to metabolic disorders, oxidative stress, and changes in cell morphology. Investigations of the appropriate doses and forms of selenium in the culture medium are crucial to maintaining cellular homeostasis in yeast.

Various biotechnological strategies exist to optimize the bioconversion of inorganic selenium in culture media [21,50,51,52]. These depend absolutely on the culture conditions (temperature, pH, nutrients, etc.), the microorganism used, and the concentrations of inorganic selenium in the experimental medium, since these all have a considerable impact on biomass [47], the intracellular selenium content [53], the amino acid and fatty acid profiles, and the pigments in the yeast [31,54]. In the present study, the nitrogen source that most favored selenium accumulation was ammonium chloride, primarily due to the absence of sulfur in the culture medium. Peptone and ammonium sulfate are nitrogen sources that, possessing sulfur, generate competition with selenium. It is known that the metabolism of selenium is analogous to that of sulfur, as in both cases, the same enzymes and proteins are used in their transport and accumulation in the cytoplasm [55]. Regarding pH, it was found that in *R. mucilaginosa* 6S, low values (4.5–5.5) favor selenium accumulation. Other authors [56] determined that in *R. glutinis* X-20, selenium accumulation is highly dependent on pH; in particular, pH 7 favors selenium accumulation in this strain, unlike what has been established in other studies where selenium accumulation was reported to be favorable at an acidic pH [57]

Thus, strains of *C. utilis* and *Yarrowia lipolytica* have been used increasingly in biotechnological studies with promising results, with yields of up to 3000 μg Se/g of dry yeast [58]. Other microorganisms have also been reported with the ability to incorporate inorganic selenium into their cell structures, such as filamentous fungi [59], bacteria of the genus *Enterobacter* [60], and red yeasts like *R. mucilaginosa*-13B [21]. Studies have been conducted on the optimization of organic selenium bioaccumulation, considering different concentrations of NaSe in the culture medium, initial pH variation, different times of inoculation of inorganic selenium, and cultivation time [43]. In the present study, by applying a strategy of NaSe incorporation (30 mg/L) in the exponential phase, it was possible to obtain 2.2 mg total Se/g biomass in *R. mucilaginosa* 6S after 48 h. This strategy led to the generation of biomass in the initial stage and, subsequently, the accumulation of selenium in the cell. Very high concentrations of NaSe had a strong inhibitory effect on the growth of this yeast.

For its part, *Rhodotorula rubra* has been studied by Pankiewicz et al. [20], who optimized selenium absorption into yeast biomass by electroporation, observing that the variable with the greatest effect on the accumulation of this element was the concentration of inorganic selenium in the culture medium. The effect of Se-enriched medium on the production of carotenoid pigments in two yeasts with high antioxidant potential, *Rhodotorula glutinis* and *Sporobolomyces roseu*, has also been evaluated [61]. It was found that the preparation of a formula of this type (yeast rich in selenium and pigments) can only be obtained by culture in two stages, the first to optimize the production of carotenoid pigments and the second for the absorption of selenium into the cells.

Just as the selenium concentration affects pigment production in yeasts, it has been established that it can also alter the fatty acid profile. It has been determined that the quantitative composition of fatty acids in yeast cells is subject to the influence of various environmental factors, including the presence of minerals or the appearance of stress conditions [31]. Under specific stress parameters in the culture, many oleaginous yeasts, including those of the genus *Rhodotorula*, begin to produce lipids because although cell division, protein synthesis, and nucleic acid synthesis all cease, the production of intracellular lipids continues. These are then stored in lipid bodies, principally as triacylglycerols [62]. Maldonade et al. [63] reported that the maximum carotenoid production by a strain of *R. mucilaginosa*, isolated from the Brazilian ecosystem, was 745 μg carotenoids/L. In *R. mucilaginosa* 6S (this study), a decrease in carotenoid content was observed when NaSe (30 mg/L) was incorporated from the initial growth phase, with a final yield of 63 μg carotenoids/g biomass, unlike when sodium selenite was added in the exponential growth phase. In this case, 163 μg carotenoids/g was achieved for *R. mucilaginosa* 6S, similar to the situation in the control used (yeast cultured without NaSe) with a value of 220 μg carotenoids/g biomass. Authors like [32] indicated that the accumulation of carotenoid pigments in *R. mucilaginosa* increases when cultured in a NaSe concentration from 5 mg/L to 20 mg/L, from 193.06 μg carotenoids/g biomass to 343 μg carotenoids/g, respectively. Likewise, it has been described that fatty acid profiles in yeasts are affected by the presence of selenium in the culture medium. According to [54], red yeasts cultured in a medium supplemented with selenium (1.2 mM) produced a large amount of linoleic and linolenic acid. In yeasts of the genus *Rhodotorula*, selenium stimulates the biosynthesis of C18 fatty acids and promotes distribution in the membrane lipids [21]. In the present study, the yeast *R. mucilaginosa* 6S presented very different fatty acid profiles when NaSe was added from the beginning of the culture or after 48 h. In the first case, PUFAs reached 43.5%, where 31.5% corresponded to linoleic acid (C18:2 n-6) and 11% to linolenic acid (C18:3n3). In contrast, with the addition of NaSe after 48 h of culture, PUFAs decreased to 8.17%, and MUFAs increased to 69.5%, mainly composed of oleic acid (C18:1n9c) (67%). Authors like [64] studied the effect of selenium on the yeast *Y. lipolytica*, observing an increase in the unsaturation index in strain ALE_70, reflected in the high values of linoleic acid (C18:2 n-6). Likewise, they pointed out that selenium influences the activity of saturases and desaturases. This situation was clearly observed here when *R. mucilaginosa* 6S was cultured under two cultivation strategies. Although NaSe concentrations were the same in both strategies, the growth phase in which it was added is clearly a key factor in determining the fatty acid profile. Research has shown that selenium has a significant impact on fatty acid profiles, cellular responses to oxidative stress, and lipid peroxidation [65]. Studies have demonstrated that selenium supplementation can increase unsaturated fatty acids in yeast biomass. Selenium has also been linked to the activation of fatty acid unsaturation, particularly in phosphatidylcholine, by raising the levels of linoleic and linolenic acids [48,54]. Research on yeast strains like *Sporidiobolus* and *Saccharomyces cerevisiae* has shown that under conditions of polyunsaturated fatty acid (PUFA) accumulation, there is a down-regulation of components of the electron transport chain in mitochondria, an up-regulation of the pentose-phosphate pathway and fatty acid β-oxidation at the transcriptional level, and alterations in enzymatic antioxidants like catalase and glutathione-S-transferase [66]. Additionally, changes in lipid metabolism, particularly fatty acid synthesis, can impact the expression of stress-response genes through histone acetylation, ultimately aiding in cellular resistance to oxidative stress and maintenance of redox homeostasis [45]. Peroxisomes, essential organelles involved in fatty acid oxidation, also rely on efficient antioxidant mechanisms to ensure proper metabolite transport and oxidation processes [45,67].

More recently, Kieliszek et al. [48] analyzed the effect of selenium on the cell composition and the amino acid and fatty acid profiles of the yeasts *S. cerevisiae* MYA-2200 and *C. utilis* ATCC 9950 when cultured with industrial waste (waste potato water and glycerol). They observed that at a dose of 20 mg Se^4+^/L in the aqueous solution, the differentiation of cell morphology was affected, and that both strains enriched with selenium also contained a large quantity of glutamic acid, aspartic acid, lysine, and leucine. In the same study, there was an increase of unsaturated fatty acids (for example, C18:1) in *S. cerevisiae*, in contrast to the *C. utilis* biomass, which contained principally margaric acid (C17:0) and hexadecanoic acid (C17:1). Another interesting aspect observed during the cultivation of *R. mucilaginosa* 6S was the color changes in the experiments, where reddish hues were observed depending on the concentration of NaSe in the culture medium. This phenomenon has been described as a response to selenium binding by yeast, which decreases with a high content of sulfur and heavy metals, and the presence of glucose can lead to a reduction of selenium in the form of SeO_3_^2−^ ions, resulting in the formation of elemental red selenium [55,68]. This elemental selenium in yeast species, such as *Candida tropicalis*, *Cryptococcus demina*, *Rhodosporidium diobovatum*, *Pichia capsulata*, *Pichia fermentans*, *Y. lipolytica*, *Rhodotorula glutinis*, and *R. mucilaginosa*, has been reported to be capable of synthesizing different nanoparticles [69].

Proteomic studies in *R. mucilaginosa* have mainly focused on analyzing its resistance to heavy metals and carotenoid production [70,71,72]. Few “omic” approaches have determined the role of proteins in other yeasts. The biotransformation of selenium involved the participation of a greater number of molecular functions. Among the molecular pathways with the greatest presence of proteins, the functions related to the binding to purine ribonucleoside triphosphate stand out, which is consistent with the greater catalytic activity of the biotransformation process (Figure S1). According to Kieliszek [73], this involves the reduction of selenium and subsequently its transformation into SeMet, processes in which a series of enzymes participate that need intermediate molecules composed of purines, such as the ATP molecule, to carry out their catalytic activity.

In the case of yeasts without selenium incorporation, the only molecular functions determined were in proteins related to oxidoreductase activity and catalytic activity, processes that were also identified in the study by [74], who observed a high antioxidant capacity and an efficient response to oxidative stress. These results could be governed by the ability of *R. mucilaginosa* 6S to synthesize carotenoids which are very effective antioxidants [32,75].

*R. mucilaginosa* is also capable of synthesizing a variety of secondary metabolites with biological activity, such as carotenoids, fatty acids, and antioxidant compounds, through metabolic pathways involving reactions catalyzed by enzymes. The oxido-reductase activity in *R. mucilaginosa* may be involved in the transformation of selenium into its elemental form. This could occur through reduction processes where oxido-reductases catalyze the conversion of selenium compounds into elemental selenium, which is a more stable and less toxic form. *Rhodotorula* species have shown considerable potential in animal nutrition due to their capacity for synthesizing beneficial biomolecules such as carotenoids, lipids, and proteins. These microorganisms can be harnessed for bioconversion to yield carotenoid-rich biomass, offering essential nutritional elements for animals [76]. Moreover, R. mucilaginosa has been shown to improve the immune functionality and gut microbiota, increasing beneficial bacteria like *Firmicutes* and *Lactobacillus* while diminishing the presence of detrimental bacteria such as Bacteroidetes [19,77]. The utilization of *Rhodotorula* yeasts, specifically *R. mucilaginosa* and *R. glutinis*, has exhibited significant promise in augmenting the nutritional composition of livestock feed by elevating the provision of vital minerals and vitamins. Through the fermentation of tofu whey wastewater, Rhodotorula species have been able to boost the zinc content by 62.3% and enhance the levels of essential vitamins B1, B2, and B6, which play a critical role in animal nutrition [78]. Overall, the diverse metabolic proficiencies and adaptability of *Rhodotorula* yeasts position them as a valuable asset for enhancing mineral provision and promoting the health and productivity of farm animals.

## 5. Conclusions

Through the optimization of cultivation conditions, it was possible to obtain 0.2529 mg Se/g of *R. mucilaginosa*, using a strategy of incorporating NaSe at 48 h of cultivation. In response to selenium exposure by the cell, it was observed, through the yeast proteome, how the metabolic machinery enables it to carry out detoxification processes evidenced by the presence of proteins associated with catalysis and redox reactions. The biomass obtained presented a reddish hue, indicative of the reduction of NaSe to elemental selenium. It was also found that selenium could have some implications in increasing the percentage of lipids, affecting the fatty acid profile towards the generation of MUFA and PUFA. Further studies are required to elucidate the aforementioned processes. Therefore, *R. mucilaginosa* 6S appears to be a promising organism for the production of selenium-enriched biomass, which can be utilized in various biotechnological applications, including in animal nutrition and aquaculture.

## Figures and Tables

**Table 1:** Biomass and content of *R. mucilaginosa* 6S cultured with 30 mg/L NaSe.

Parameters	Control	Rm (t = 0)	Rm (t = 48)
*Biomass* (g/L)	7.4 ± 0.0	1.0 ± 0.1 *	4.5 ± 0.1 *
*Total Lipid* (%)	3.2 ± 0.1	3.4 ± 0.1	4.5 ± 0.0 *
*SAFA* (%)	47.6 ± 0.4	26.9 ± 1.5 *	22.4 ± 0.5 *
*MUFA* (%)	24.0 ± 1.1	29.6 ± 0.9 *	69.5 ± 0.6 *
*PUFA* (%)	29.6 ± 0.5	43.6 ± 0.3 *	8.2 ± 0.2 *
*Protein* (%)	18.2 ± 0.5	16.9 ± 1.2	17.7 ± 0.7
*Carotenoids* (µg/g biomass)	221.3 ± 22.6	63.3 ± 6.0 *	163.3 ± 8.3 *
*Selenium* (mg/g biomass)	0.1 ± 0.0	0.2 ± 0.0	2.5 ± 0.4 *

Control = *R. mucilaginosa* 6S grown without sodium selenite. Rm (t = 0) = *R. mucilaginosa* 6S grown with sodium selenite since the initial phase (0 h). Rm (t = 48) = *R. mucilaginosa* 6S grown with sodium selenite since the exponential phase (48 h). (*) represents significant differences with respect to the control with *p*–value < 0.05.

**Table 2:** Pearson’s correlation for NaSe addition time treatments and the parameters evaluated.

Parameters	Treatments	Selenium
*Biomass* (g/L)	−0.462	0.022
*Total Lipid* (%)	0.902 *	0.980 *
*SAFA* (%)	−0.935 *	−0.647
*MUFA* (%)	0.916 *	0.983 *
*PUFA* (%)	−0.599	−0.900 *
*Protein* (%)	−0.227	0.108
*Carotenoids* (µg/g biomass)	−0.357	0.131
*Selenium* (mg/g biomass)	0.869 *	1

(*) represents significant Pearson correlation with *p*-value < 0.05.

## Data Availability

The data presented in this study are available in this article.

## Supplementary Materials

The following supporting information can be downloaded at: https://www.mdpi.com/article/10.3390/biom14060629/s1, Figure S1: (A) Molecular function of proteins significantly overexpressed due to selenium treatment. (B) Signaling pathways originating from proteins significantly overexpressed in the control sample.
