# Supplementary material for: Enhancing Selenium Accumulation in Rhodotorula mucilaginosa Strain 6S Using a Proteomic Approach for Aquafeed Development
Source: Biomolecules. 2024 May 27;14(6):629. doi: 10.3390/biom14060629 (PMC11201420; doi:10.3390/biom14060629)
Supplement: Supplementary file 1 [file biomolecules-14-00629-s001.zip › biomolecules-3018104-supplementary.pdf]

Supplementary Material

A

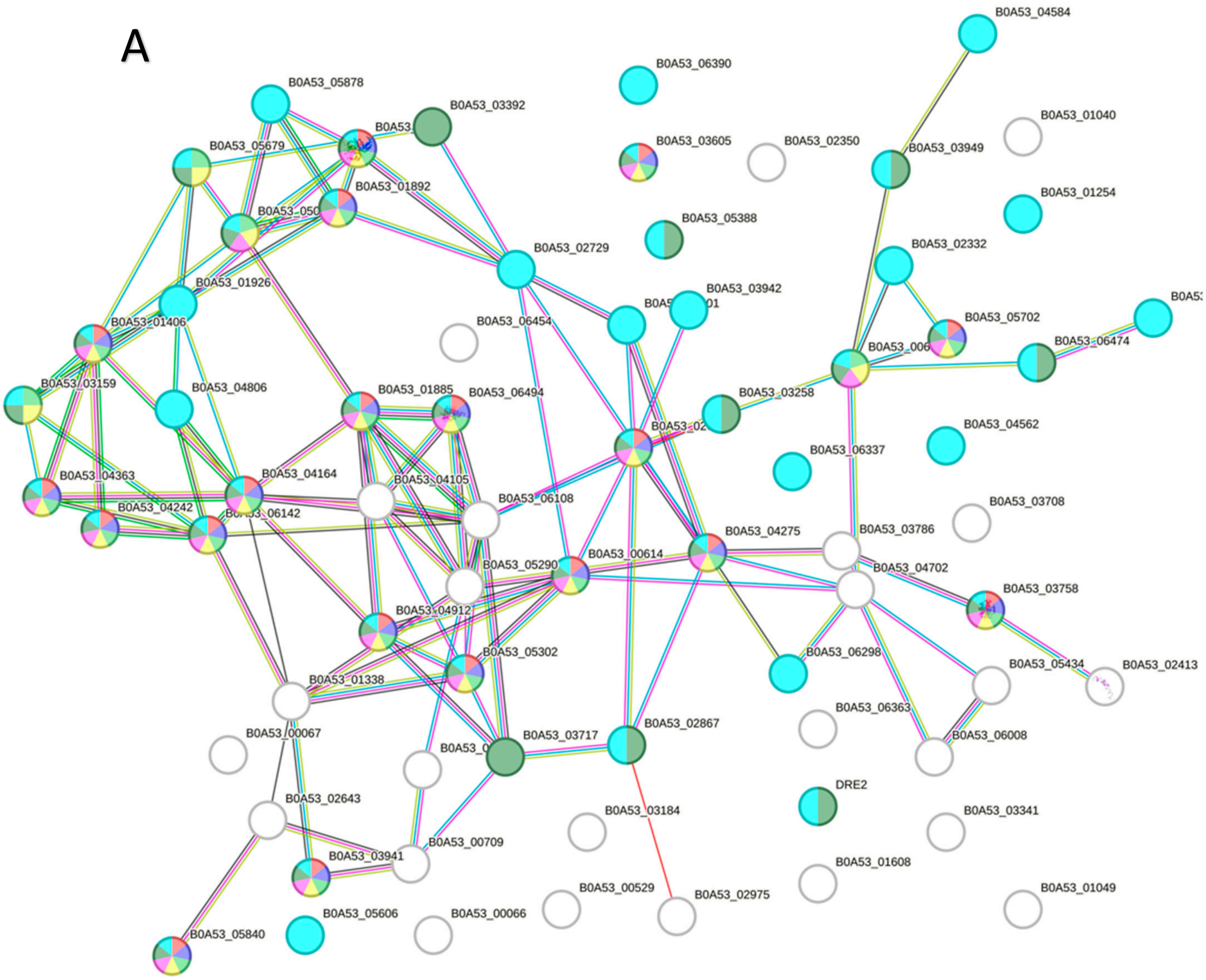

Molecular Function (Gene Ontology)

GO-term	description	count in network	strength	false discovery rate
GO:0035639	Purine ribonucleoside triphosphate binding	19 of 649	0.42	0.0318
GO:0032555	Purine ribonucleotide binding	19 of 655	0.42	0.0318
GO:0043168	Anion binding	23 of 821	0.4	0.0225
GO:0036094	Small molecule binding	23 of 889	0.37	0.0318
GO:0000166	Nucleotide binding	21 of 823	0.36	0.0318
GO:0043167	Ion binding	31 of 1526	0.26	0.0318
GO:0003824	Catalytic activity	44 of 2455	0.21	0.0318

B

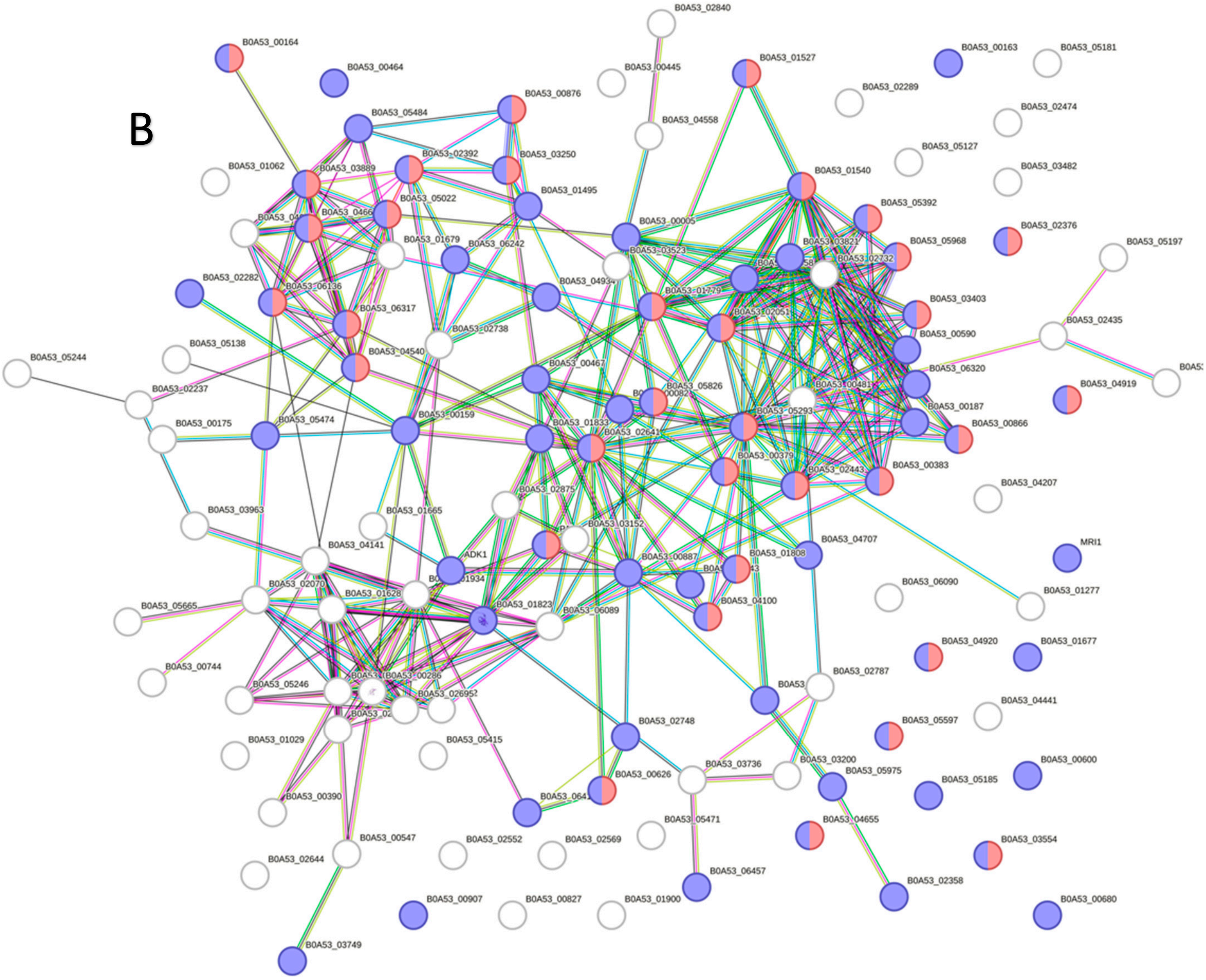

Molecular Function (Gene Ontology)					
GO-term	description	count in network	strength	false discovery rate	
GO:0016491	Oxidoreductase activity	34 of 472	0.56	5.53e-08	●
GO:0003824	Catalytic activity	70 of 2455	0.16	0.0470	●

Figura S1. (A) Molecular function of proteins significantly overexpressed due to selenium treatment. Of the 133 proteins identified as overexpressed, 71 were identified in the database available for Rhodotorula spp. through the STRING platform for signaling pathway analysis. Lines indicate interaction, circles denote the identified proteins with their respective names, and colors indicate the molecular function. (B) Signaling pathways originating from proteins significantly overexpressed in the control sample. Of the 178 proteins identified as overexpressed, 126 were identified in the available database for *Rhodotorula* spp. through the STRING platform for signaling pathway analysis. Lines indicate interaction, circles denote the identified proteins with their respective names, and colors indicate the molecular function as indicated in the table below.
